# Supplementary material for: A microbial safari: finding evidence of Mycobacterium bovis DNA in soil from the Kruger National Park, South Africa
Source: Microbiol Spectr. 2025 Dec 3;14(1):e01658-25. doi: 10.1128/spectrum.01658-25 (PMC12772276; doi:10.1128/spectrum.01658-25)
Supplement: Supplemental tables — Tables S1 and S2. [file spectrum.01658-25-s0002.docx]

**Table S1**. Quality and quantity of DNA extracted from a subset of soil samples collected near water sources at six different locations in the Kruger National Park (KNP), South Africa.

| Sample Information^a^ | | | Quantity and Quality Testing^b^ | | |
| --- | --- | --- | --- | --- | --- |
| No. | Date | Location | Concentration | DNA Presence | *16S* PCR |
| 5 | 8/9th | 1 | 138 | Present | Amplified |
| 12 | 8/9th | 3 | 44 | Present | Amplified |
| 20 | 8/9th | 5 | 79 | Present | Amplified |
| 29 | 8/9th | 6 | 88 | Present | Amplified |
| 41 | 13/14th | 3 | 117 | Present | NA |
| 48 | 13/14th | 4 | 214 | Present | Amplified |
| 53 | 13/14th | 5 | 186 | Present | Amplified |
| 69 | 15/16th | 2 | 82 | Present | Amplified |
| 89 | 15/16th | 6 | 273 | Present | Amplified |
| 93 | 20/21st | 1 | 56 | Present | NA |
| 111 | 20/21st | 5 | 43 | Present | Amplified |
| 112 | 20/21st | 5 | 121 | Present | Amplified |
| 118 | 20/21st | 6 | 201 | Present | Amplified |
| 142 | 23/24th | 5 | 63 | Present | Amplified |
| 161 | 28/29th | 3 | 136 | Present | Amplified |
| 162 | 28/29th | 3 | 92 | Present | Amplified |
| 169 | 28/29th | 4 | 103 | Present | Amplified |
| 175 | 28/29th | 6 | 167 | Present | Amplified |

^a^A subset of 18 DNA samples extracted from Kruger National Park soil were selected using a random number generator (Furey, 2023) from a total of 180 samples. The sample number (No.), date of collection in June 2022 and the location were recorded. The co-ordinates (latitude and longitude) were -25.023 and 31.526 (site 1), -25.045 and 31.570 (site 2), -25.147 and 31.556 (site 3), -25.255 and 31.845 (site 4), -25.214 and 31.923(site 5) and -25.117 and 31.913(site 6), respectively.

^b^The mean concentration (ng/μL) of extracted DNA was determined using Qubit spectrophotometry, according to the manufacturer’s instructions. DNA was diluted (1:10) and gel electrophoresis was used to confirm if DNA bands were present or absent and if *16S* PCR resulted in no amplification (NA) or amplified the expected 1550 bp gene region.

**Table S2**. The DNA from soil samples^a^ collected near water sources at six different locations in the Kruger National Park, South Africa, were screened for *Mycobacterium* based on *hsp65* polymerase chain reaction (PCR) and Sanger amplicon sequencing (SAS)^b^.

|  | Sample Information^a^ | | | | Sanger Amplicon Sequencing (SAS)^b^ | | | | | | |
| --- | --- | --- | --- | --- | --- | --- | --- | --- | --- | --- | --- |
| No. | Date | Location | | PCR | | Sanger Result | | P_C_ | | | P_IM_ |
| 1 | 8/9^th^ | 1 | | Amplified | | UA | | UA | | | UA |
| 2 | 8/9^th^ | 1 | | Amplified | | No *Mycobacteria* | | 78 | | | 88 |
| 3 | 8/9^th^ | 1 | | Amplified | | *M. psychrotolerans* | | 100 | | | 90 |
| 4 | 8/9^th^ | 1 | | Amplified | | *M. psychrotolerans* | | 100 | | | 90 |
| 5* | 8/9^th^ | 1 | | Amplified | | *M. chlorophenolicum* | | 100 | | | 90 |
| 6 | 8/9^th^ | 2 | | NA | | NA | | NA | | | NA |
| 7 | 8/9^th^ | 2 | | Amplified | | No *Mycobacteria* | | 97 | | | 86 |
| 8 | 8/9^th^ | 2 | | NA | | NA | | NA | | | NA |
| 9 | 8/9^th^ | 2 | | NA | | NA | | NA | | | NA |
| 10 | 8/9^th^ | 2 | | NA | | NA | | NA | | | NA |
| 11 | 8/9^th^ | 3 | | Amplified | | No *Mycobacteria* | | 100 | | | 89 |
| 12* | 8/9^th^ | 3 | | Amplified | | No *Mycobacteria* | | 100 | | | 86 |
| 13 | 8/9^th^ | 3 | | Amplified | | No *Mycobacteria* | | 98 | | | 88 |
| 14 | 8/9^th^ | 3 | | Amplified | | No *Mycobacteria* | | 100 | | | 90 |
| 15 | 8/9^th^ | 3 | | NA | | NA | | NA | | | NA |
| 16 | 8/9^th^ | 4 | | NA | | NA | | NA | | | NA |
| 17 | 8/9^th^ | 4 | | Amplified | | *M. tusciae* | | 100 | | | 90 |
| 18 | 8/9^th^ | 4 | | Amplified | | No *Mycobacteria* | | 100 | | | 88 |
| 19 | 8/9^th^ | 4 | | Amplified | | No *Mycobacteria* | | 98 | | | 83 |
| 20* | 8/9^th^ | 4 | | Amplified | | *M. psychrotolerans* | | 100 | | | 90 |
| 21 | 8/9^th^ | 5 | | Amplified | | UA | | UA | | | UA |
| 22 | 8/9^th^ | 5 | | Amplified | | No *Mycobacteria* | | 96 | | | 81 |
| 23 | 8/9^th^ | 5 | | Amplified | | UA | | UA | | | UA |
| 24 | 8/9^th^ | 5 | | Amplified | | No *Mycobacteria* | | 100 | | | 87 |
| 25 | 8/9^th^ | 5 | | NA | | NA | | NA | | | NA |
| 26 | 8/9^th^ | 6 | | Amplified | | No *Mycobacteria* | | 99 | | | 90 |
| 27 | 8/9^th^ | 6 | | Amplified | | No *Mycobacteria* | | 100 | | | 91 |
| 28 | 8/9^th^ | 6 | | Amplified | | No *Mycobacteria* | | 99 | | | 92 |
| 29* | 8/9^th^ | 6 | | Amplified | | No *Mycobacteria* | | 100 | | | 92 |
| 30 | 8/9^th^ | 6 | | Amplified | | No *Mycobacteria* | | 100 | | | 89 |
| 31 | 3/14^th^ | 1 | | NA | | NA | | NA | | | NA |
| 32 | 3/14^th^ | 1 | | Amplified | | **MTBC** | | 100 | | | 98 |
| 33 | 3/14^th^ | 1 | | Amplified | | No *Mycobacteria* | | 100 | | | 96 |
| 34 | 3/14^th^ | 1 | | Amplified | | No *Mycobacteria* | | 99 | | | 90 |
| 35 | 3/14^th^ | 1 | | Amplified | | No *Mycobacteria* | | 99 | | | 90 |
| 36 | 3/14^th^ | 2 | | Amplified | | No *Mycobacteria* | | 98 | | | 90 |
| 37 | 3/14^th^ | 2 | | NA | | NA | | NA | | | NA |
| 38 | 3/14^th^ | 2 | | NA | | NA | | NA | | | NA |
| 39 | 3/14^th^ | 2 | | NA | | NA | | NA | | | NA |
| 40 | 3/14^th^ | 2 | | Amplified | | *Mycobacterium sp.* | | 100 | | | 89 |
| 41* | 3/14^th^ | 3 | | NA | | NA | | NA | | | NA |
| 42 | 3/14^th^ | 3 | | Amplified | | UA | | UA | | | UA |
| 43 | 3/14^th^ | 3 | | NA | | NA | | NA | | | NA |
| 44 | 3/14^th^ | 3 | | Amplified | | *Mycobacterium sp.* | | 100 | | | 89 |
| 45 | 3/14^th^ | 3 | | Amplified | | No *Mycobacteria* | | 98 | | | 91 |
| 46 | 3/14^th^ | 4 | | Amplified | | No *Mycobacteria* | | 99 | | | 89 |
| 47 | 3/14^th^ | 4 | | Amplified | | *Mycobacterium sp.* | | 100 | | | 86 |
| 48* | 3/14^th^ | 4 | | Amplified | | *Mycobacterium sp.* | | 100 | | | 89 |
| 49 | 3/14^th^ | 4 | | Amplified | | *M. chubuense* | | 100 | | | 91 |
| 50 | 3/14^th^ | 4 | | Amplified | | No *Mycobacteria* | | 99 | | | 89 |
| 51 | 3/14^th^ | 5 | | NA | | NA | | NA | | | NA |
| 52 | 3/14^th^ | 5 | | NA | | NA | | NA | | | NA |
| 53* | 3/14^th^ | 5 | | Amplified | | UA | | UA | | | UA |
|  |  |  | |  | |  | |  | | |  |
| Sample Information^a^ | | | | Sanger Amplicon Sequencing (SAS)^b^ | | | | | | | |
| No. | Date |  | | PCR | | Sanger Result | | P_C_ | | | P_IM_ |
| 54 | 3/14^th^ | 5 | | NA | | NA | | NA | | | NA |
| 55 | 3/14^th^ | 5 | | NA | | NA | | NA | | | NA |
| 56 | 3/14^th^ | 6 | | Amplified | | *Mycobacterium sp.* | | 100 | | | 87 |
| 57 | 3/14^th^ | 6 | | Amplified | | *Mycobacterium sp.* | | 100 | | | 91 |
| 58 | 3/14^th^ | 6 | | NA | | NA | | NA | | | NA |
| 59 | 3/14^th^ | 6 | | NA | | NA | | NA | | | NA |
| 60 | 13/14^th^ | 6 | | NA | | NA | | NA | | | NA |
| 61 | 15/16^th^ | 1 | Amplified | | | | *M. psychrotolerans* | | *98* | 90 | |
| 62 | 15/16^th^ | 1 | Amplified | | | | *M. novocastrense* | | 99 | 93 | |
| 63 | 15/16^th^ | 1 | Amplified | | | | No *Mycobacteria* | | 94 | 84 | |
| 64 | 15/16^th^ | 1 | Amplified | | | | *Mycobacterium sp.* | | 99 | 90 | |
| 65 | 15/16^th^ | 1 | NA | | | | NA | | NA | NA | |
| 66 | 15/16^th^ | 2 | Amplified | | | | No *Mycobacteria* | | 100 | 91 | |
| 67 | 15/16^th^ | 2 | Amplified | | | | No *Mycobacteria* | | 100 | 93 | |
| 68 | 15/16^th^ | 2 | Amplified | | | | No *Mycobacteria* | | 100 | 90 | |
| 69* | 15/16^th^ | 2 | Amplified | | | | No *Mycobacteria* | | 100 | 91 | |
| 70 | 15/16^th^ | 2 | NA | | | | NA | | NA | NA | |
| 71 | 15/16^th^ | 3 | Amplified | | | | **MTBC** | | 99 | 99 | |
| 72 | 15/16^th^ | 3 | NA | | | | NA | | NA | NA | |
| 73 | 15/16^th^ | 3 | NA | | | | NA | | NA | NA | |
| 74 | 15/16^th^ | 3 | Amplified | | | | No *Mycobacteria* | | 100 | 87 | |
| 75 | 15/16^th^ | 3 | Amplified | | | | **MTBC** | | 100 | 96 | |
| 76 | 15/16^th^ | 4 | Amplified | | | | No *Mycobacteria* | | 100 | 92 | |
| 77 | 15/16^th^ | 4 | Amplified | | | | *M. kubicae* | | 99 | 90 | |
| 78 | 15/16^th^ | 4 | Amplified | | | | MAC | | 99 | 90 | |
| 79 | 15/16^th^ | 4 | Amplified | | | | MAC | | 100 | 90 | |
| 80 | 15/16^th^ | 4 | Amplified | | | | No *Mycobacteria* | | 94 | 84 | |
| 81 | 15/16^th^ | 5 | Amplified | | | | No *Mycobacteria* | | 100 | 84 | |
| 82 | 15/16^th^ | 5 | Amplified | | | | No *Mycobacteria* | | 100 | 84 | |
| 83 | 15/16^th^ | 5 | Amplified | | | | *Mycobacterium sp.* | | 100 | 88 | |
| 84 | 15/16^th^ | 5 | Amplified | | | | No *Mycobacteria* | | 99 | 80 | |
| 85 | 15/16^th^ | 5 | Amplified | | | | No *Mycobacteria* | | 99 | 90 | |
| 86 | 15/16^th^ | 6 | Amplified | | | | *Mycobacterium sp.* | | 99 | 82 | |
| 87 | 15/16^th^ | 6 | Amplified | | | | No *Mycobacteria* | | 100 | 89 | |
| 88 | 15/16^th^ | 6 | NA | | | | NA | | NA | NA | |
| 89* | 15/16^th^ | 6 | Amplified | | | | No *Mycobacteria* | | 99 | 82 | |
| 90 | 15/16^th^ | 6 | Amplified | | | | No *Mycobacteria* | | 99 | 90 | |
| 91 | 20/21^st^ | 1 | NA | | | | NA | | NA | NA | |
| 92 | 20/21^st^ | 1 | NA | | | | NA | | NA | NA | |
| 93* | 20/21^st^ | 1 | NA | | | | NA | | NA | NA | |
| 94 | 20/21^st^ | 1 | NA | | | | NA | | NA | NA | |
| 95 | 20/21^st^ | 1 | NA | | | | NA | | NA | NA | |
| 96 | 20/21^st^ | 2 | NA | | | | NA | | NA | NA | |
| 97 | 20/21^st^ | 2 | NA | | | | NA | | NA | NA | |
| 98 | 20/21^st^ | 2 | Amplified | | | | No *Mycobacteria* | | 99 | 82 | |
| 99 | 20/21^st^ | 2 | NA | | | | NA | | NA | NA | |
| 100 | 20/21^st^ | 2 | Amplified | | | | No *Mycobacteria* | | 98 | 89 | |
| 101 | 20/21^st^ | 3 | NA | | | | NA | | NA | NA | |
| 102 | 20/21^st^ | 3 | Amplified | | | | No *Mycobacteria* | | 99 | 82 | |
| 103 | 20/21^st^ | 3 | Amplified | | | | No *Mycobacteria* | | 98 | 89 | |
| 104 | 20/21^st^ | 3 | Amplified | | | | No *Mycobacteria* | | 99 | 84 | |
| 105 | 20/21^st^ | 3 | Amplified | | | | No *Mycobacteria* | | 99 | 87 | |
| 106 | 20/21^st^ | 4 | Amplified | | | | No *Mycobacteria* | | 100 | 91 | |
| 107 | 20/21^st^ | 4 | Amplified | | | | *M. asiaticum* | | 100 | 91 | |
| 108 | 20/21^st^ | 4 | Amplified | | | | No *Mycobacteria* | | 100 | 87 | |
| 109 | 20/21^st^ | 4 | Amplified | | | | MAC | | 100 | 91 | |
| 110 | 20/21^st^ | 4 | Amplified | | | | *Mycobacterium* sp. | | 98 | 89 | |
| 111* | 20/21^st^ | 5 | Amplified | | | | No *Mycobacteria* | | 99 | 90 | |
| 112* | 20/21^st^ | 5 | Amplified | | | | No *Mycobacteria* | | 99 | 87 | |
| 113 | 20/21^st^ | 5 | Amplified | | | | *Mycobacterium sp.* | | 100 | 88 | |
| 114 | 20/21^st^ | 5 | Amplified | | | | No *Mycobacteria* | | 96 | 90 | |
| 115 | 20/21^st^ | 5 | NA | | | | NA | | NA | NA | |
| 116 | 20/21^st^ | 6 | Amplified | | | | *Mycobacterium sp.* | | 100 | 88 | |
| 117 | 20/21^st^ | 6 | Amplified | | | | No *Mycobacteria* | | 97 | 88 | |
| 118* | 20/21^st^ | 6 | Amplified | | | | No *Mycobacteria* | | 100 | 87 | |
| Sample Information^a^ | | | Sanger Amplicon Sequencing (SAS)^b^ | | | | | | | | |
| No. | Date | Location | PCR (*hsp65*) | | | | Sanger Result | | P_C_ | P_IM_ | |
| 119 | 20/21^st^ | 6 | Amplified | | | | *Mycobacterium sp.* | | 100 | 86 | |
| 120 | 20/21^st^ | 6 | Amplified | | | | No *Mycobacteria* | | 100 | 88 | |
| 121 | 23/24^th^ | 1 | Amplified | | | | No *Mycobacteria* | | 99 | 87 | |
| 122 | 23/24^th^ | 1 | Amplified | | | | *M. komanii* | | 100 | 90 | |
| 123 | 23/24^th^ | 1 | Amplified | | | | *M. rutilum* | | 100 | 90 | |
| 124 | 23/24^th^ | 1 | NA | | | | NA | | NA | NA | |
| 125 | 23/24^th^ | 1 | Amplified | | | | *Mycobacterium sp.* | | 100 | 86 | |
| 126 | 23/24^th^ | 2 | Amplified | | | | No *Mycobacteria* | | 98 | 92 | |
| 127 | 23/24^th^ | 2 | Amplified | | | | No *Mycobacteria* | | 100 | 93 | |
| 128 | 23/24^th^ | 2 | Amplified | | | | No *Mycobacteria* | | 100 | 91 | |
| 129 | 23/24^th^ | 2 | NA | | | | NA | | NA | NA | |
| 130 | 23/24^th^ | 2 | Amplified | | | | No *Mycobacteria* | | 96 | 91 | |
| 131 | 23/24^th^ | 3 | Amplified | | | | No *Mycobacteria* | | 100 | 89 | |
| 132 | 23/24^th^ | 3 | Amplified | | | | No *Mycobacteria* | | 100 | 90 | |
| 133 | 23/24^th^ | 3 | Amplified | | | | No *Mycobacteria* | | 99 | 91 | |
| 134 | 23/24^th^ | 3 | Amplified | | | | No *Mycobacteria* | | 100 | 88 | |
| 135 | 23/24^th^ | 3 | Amplified | | | | No *Mycobacteria* | | 100 | 92 | |
| 136 | 23/24^th^ | 4 | Amplified | | | | No *Mycobacteria* | | 99 | 89 | |
| 137 | 23/24^th^ | 4 | Amplified | | | | *Mycobacterium sp.* | | 99 | 92 | |
| 138 | 23/24^th^ | 4 | Amplified | | | | *Mycobacterium sp.* | | 100 | 88 | |
| 139 | 23/24^th^ | 4 | NA | | | | NA | | NA | NA | |
| 140 | 23/24^th^ | 4 | NA | | | | NA | | NA | NA | |
| 141 | 23/24^th^ | 5 | Amplified | | | | No *Mycobacteria* | | 99 | 86 | |
| 142* | 23/24^th^ | 5 | Amplified | | | | No *Mycobacteria* | | 100 | 91 | |
| 143 | 23/24^th^ | 5 | NA | | | | NA | | NA | NA | |
| 144 | 23/24^th^ | 5 | Amplified | | | | *M. psychrotolerans* | | 100 | 91 | |
| 145 | 23/24^th^ | 5 | Amplified | | | | No *Mycobacteria* | | 99 | 80 | |
| 146 | 23/24^th^ | 6 | Amplified | | | | *M. chubuense* | | 100 | 92 | |
| 147 | 23/24^th^ | 6 | Amplified | | | | No *Mycobacteria* | | 100 | 91 | |
| 148 | 23/24^th^ | 6 | Amplified | | | | No *Mycobacteria* | | 98 | 90 | |
| 149 | 23/24^th^ | 6 | Amplified | | | | No *Mycobacteria* | | 100 | 87 | |
| 150 | 23/24^th^ | 6 | Amplified | | | | No *Mycobacteria* | | 99 | 88 | |
| 151 | 28/29^th^ | 1 | Amplified | | | | *Mycobacterium* sp. | | 100 | 88 | |
| 152 | 28/29^th^ | 1 | Amplified | | | | *M. novocastrense* | | 99 | 91 | |
| 153 | 28/29^th^ | 1 | Amplified | | | | *M. chubuense* | | 100 | 92 | |
| 154 | 28/29^th^ | 1 | Amplified | | | | No mycobacteria | | 100 | 87 | |
| 155 | 28/29^th^ | 1 | Amplified | | | | *Mycobacterium sp.* | | 99 | 88 | |
| 156 | 28/29^th^ | 2 | NA | | | | NA | | NA | NA | |
| 157 | 28/29^th^ | 2 | Amplified | | | | No *Mycobacteria* | | 100 | 89 | |
| 158 | 28/29^th^ | 2 | Amplified | | | | No *Mycobacteria* | | 100 | 91 | |
| 159 | 28/29^th^ | 2 | NA | | | | NA | | NA | NA | |
| 160 | 28/29^th^ | 2 | Amplified | | | | No *Mycobacteria* | | 100 | 91 | |
| 161* | 28/29^th^ | 3 | Amplified | | | | No *Mycobacteria* | | 100 | 89 | |
| 162* | 28/29^th^ | 3 | Amplified | | | | No *Mycobacteria* | | 100 | 91 | |
| 163 | 28/29^th^ | 3 | Amplified | | | | No *Mycobacteria* | | 99 | 91 | |
| 164 | 28/29^th^ | 3 | Amplified | | | | No *Mycobacteria* | | 100 | 90 | |
| 165 | 28/29^th^ | 3 | Amplified | | | | No *Mycobacteria* | | 99 | 89 | |
| 166 | 28/29^th^ | 4 | Amplified | | | | No *Mycobacteria* | | 90 | 80 | |
| 167 | 28/29^th^ | 4 | Amplified | | | | MAC | | 98 | 90 | |
| 168 | 28/29^th^ | 4 | Amplified | | | | No *Mycobacteria* | | 100 | 87 | |
| 169* | 28/29^th^ | 4 | Amplified | | | | *Mycobacterium sp.* | | 100 | 87 | |
| 170 | 28/29^th^ | 4 | Amplified | | | | *Mycobacterium sp.* | | 99 | 88 | |
| 171 | 28/29^th^ | 5 | Amplified | | | | No *Mycobacteria* | | 99 | 80 | |
| 172 | 28/29^th^ | 5 | Amplified | | | | UA | | UA | UA | |
| 173 | 28/29^th^ | 5 | NA | | | | NA | | NA | NA | |
| 174 | 28/29^th^ | 5 | Amplified | | | | No *Mycobacteria* | | 99 | 83 | |
| 175* | 28/29^th^ | 5 | Amplified | | | | No *Mycobacteria* | | 99 | 90 | |
| 176 | 28/29^th^ | 6 | Amplified | | | | MAC | | 100 | 91 | |
| 177 | 28/29^th^ | 6 | Amplified | | | | No *Mycobacteria* | | 98 | 90 | |
| 178 | 28/29^th^ | 6 | Amplified | | | | No *Mycobacteria* | | 99 | 87 | |
| 179 | 28/29^th^ | 6 | Amplified | | | | No *Mycobacteria* | | 100 | 87 | |
| 180 | 28/29^th^ | 6 | Amplified | | | | No *Mycobacteria* | | 96 | 88 | |
| Pos | - |  | Amplified | | | | **MTBC** | | 100 | 100 | |
| Neg |  |  | NA | | | | UA | | UA | UA | |

^a^Sample information including sample number (no.), sample location (1-6), and the date of collection in June 2022. The location co-ordinates (latitude and longitude) were -25.023 and 31.526 (site 1), -25.045 and 31.570 (site 2), -25.147 and 31.556 (site 3), -25.255 and 31.845 (site 4), -25.214 and 31.923(site 5) and -25.117 and 31.913(site 6), respectively.

^b^Each soil sample underwent DNA extraction and *hsp65* PCR amplification. Gel electrophoresis was used to confirm if PCR resulted in no amplification (NA) or amplified the expected 439 bp gene region.

^c^If PCR was successful, amplicons were sent for Sanger amplicon sequencing (SAS). Sequences with percentage coverage (P_C_) ≥ 90% and identity match (P_IM_) ≥ 80% compared to sequences from known *Mycobacteria* were identified to *Mycobacterium* genus level. Sequences which did not meet this threshold were considered unassigned (UA). Moreover, sequences with P_c_ and P_IM_ ≥ 90% with known *Mycobacterium* species were identified to species level. Since *Mycobacterium tuberculosis* complex (**MTBC**) or *M. avium* complex (MAC) members could not be distinguished based on the *hsp65* gene target sequencing, the species complex was assigned rather than the individual subspecies/ecotype.

*A subset of 18 samples were selected for evaluating average DNA yield and quality testing.
